# Mouse Saliva Inhibits Transit of Influenza Virus to the Lower Respiratory Tract by Efficiently Blocking Influenza Virus Neuraminidase Activity

**DOI:** 10.1128/JVI.00145-17

**Published:** 2017-06-26

**Authors:** Brad Gilbertson, Wy Ching Ng, Simon Crawford, Jenny L. McKimm-Breschkin, Lorena E. Brown

**Affiliations:** a Department of Microbiology and Immunology, The University of Melbourne at the Peter Doherty Institute for Infection and Immunity, Parkville, Victoria, Australia; b Ecosystem and Forest Sciences, University of Melbourne, Parkville, Victoria, Australia; St. Jude Children's Research Hospital

**Keywords:** influenza, innate immunity, mouse model, neuraminidase

## Abstract

We previously identified a novel inhibitor of influenza virus in mouse saliva that halts the progression of susceptible viruses from the upper to the lower respiratory tract of mice *in vivo* and neutralizes viral infectivity in MDCK cells. Here, we investigated the viral target of the salivary inhibitor by using reverse genetics to create hybrid viruses with some surface proteins derived from an inhibitor-sensitive strain and others from an inhibitor-resistant strain. These viruses demonstrated that the origin of the viral neuraminidase (NA), but not the hemagglutinin or matrix protein, was the determinant of susceptibility to the inhibitor. Comparison of the NA sequences of a panel of H3N2 viruses with differing sensitivities to the salivary inhibitor revealed that surface residues 368 to 370 (N2 numbering) outside the active site played a key role in resistance. Resistant viruses contained an EDS motif at this location, and mutation to either EES or KDS, found in highly susceptible strains, significantly increased *in vitro* susceptibility to the inhibitor and reduced the ability of the virus to progress to the lungs when the viral inoculum was initially confined to the upper respiratory tract. In the presence of saliva, viral strains with a susceptible NA could not be efficiently released from the surfaces of infected MDCK cells and had reduced enzymatic activity based on their ability to cleave substrate *in vitro*. This work indicates that the mouse has evolved an innate inhibitor similar in function, though not in mechanism, to what humans have created synthetically as an antiviral drug for influenza virus.

**IMPORTANCE** Despite widespread use of experimental pulmonary infection of the laboratory mouse to study influenza virus infection and pathogenesis, to our knowledge, mice do not naturally succumb to influenza. Here, we show that mice produce their own natural form of neuraminidase inhibitor in saliva that stops the virus from reaching the lungs, providing a possible mechanism through which the species may not experience severe influenza virus infection in the wild. We show that the murine salivary inhibitor targets the outer surface of the influenza virus neuraminidase, possibly occluding entry to the enzymatic site rather than binding within the active site like commercially available neuraminidase inhibitors. This knowledge sheds light on how the natural inhibitors of particular species combat infection.

## INTRODUCTION

Influenza A virus (IAV) is an important respiratory pathogen and is the major cause of ongoing global morbidity and mortality. Innate immunity is critical in the early stages of IAV infection, limiting virus spread in host tissues before the induction of the adaptive response. There are several innate immune mechanisms that participate in the early containment of IAV, including a variety of soluble innate inhibitors that are present in respiratory secretions. They include collectins, defensins, pentraxins, mucins, and salivary scavenger cysteine-rich glycoprotein 340 (gp340) (1–8). Understanding their mechanism(s) of action is crucial if their therapeutic or prophylactic potential is to be exploited.

In the mouse model of IAV infection, it has been reported that when the viral inoculum is initially restricted to the nose during intranasal delivery to the upper respiratory tract (URT), the A/Udorn/307/72 (Udorn; H3N2) strain of influenza A virus causes a localized infection that gradually progresses to the trachea and lungs, whereas the A/Puerto Rico/8/34 (PR8; H1N1) virus causes an infection that remains almost entirely in the nasal passages (9, 10). In the accompanying paper (11), we show that following total respiratory tract (TRT) delivery (i.e., when the majority of the inoculum bypasses the oropharynx) PR8 virus replicates to high titers in the nose, trachea, and lungs and is lethal to mice at doses as low as 100 PFU. However, when the inoculum is restricted to the nose, doses as high as 10^4.5^ PFU are nonlethal and infectious virus is rarely recovered from the lungs. The difference in viral progression to the lower respiratory tract (LRT) between the Udorn and PR8 viruses was due to the relative sensitivity of the viruses to a previously undescribed innate inhibitor of influenza virus present in mouse saliva (11). Saliva secreted into the oral cavity can bathe the oropharynx, providing an opportunity for an inhibitor to stop the virus from infecting epithelial cells in the oropharynx and progressing from there along the mucosa toward the trachea and lungs. Out of 25 viral strains examined, Udorn virus and the closely related A/Port Chalmers/1/73 and A/Victoria/3/75 (all H3N2) viruses were the only strains resistant to the salivary inhibitor (11). Inhibition of PR8 virus and other susceptible strains was not negated by treatment of saliva with Vibrio cholerae or Arthrobacter ureafaciens receptor-destroying enzyme (RDE), suggesting that sialic acid, to which the viral hemagglutinin (HA) and neuraminidase (NA) bind, was not the primary determinant of sensitivity to the inhibitor.

In this study, we investigated the viral target of this inhibitor. Mixing saliva and virus together *in vitro* resulted in potent virus neutralization (11), implying that the salivary inhibitor is likely to bind to one of the viral surface proteins, namely, the HA, NA, or ion channel (M2) protein. To define the viral target of the inhibitor, we used reverse genetics to generate hybrid PR8 viruses containing either the HA, NA, or matrix protein (M) gene from Udorn virus, and we show that the Udorn NA confers resistance to the inhibitor, with residues 368 to 370 of the protein being a key determinant of susceptibility.

## RESULTS

### The salivary inhibitor of PR8 virus replication targets viral NA.

**FIG 1:**
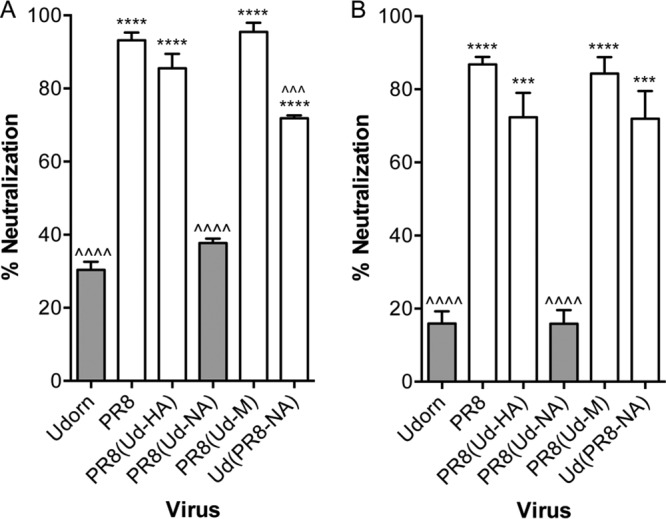
Neutralization of hybrid viruses by untreated and RDE-treated saliva. Reverse-engineered viruses (5,000 PFU) on either a PR8 or Udorn backbone were mixed with untreated (A) or RDE-treated (B) saliva at a 9:1 (vol/vol) ratio of saliva to virus. The mixtures were incubated at 37°C for 30 min and then directly assessed for the ability to form plaques in MDCK cells. The data represent the percentages of virus neutralized by saliva compared to control mixtures containing 5,000 PFU of virus and RPMI plus BSA. The means and standard deviations of the results of at least 3 individual experiments, each performed in triplicate, are shown. Viruses containing Udorn NA are represented by dark-gray bars and those containing a PR8 NA by white bars. Compared to PR8, ^^^, *P* < 0.001; and ^^^^, *P* < 0.0001. Compared to Udorn, ***, *P* < 0.001; and ****, *P* < 0.0001.

To determine the target of the murine salivary inhibitor, we used reverse genetics to create hybrid viruses expressing either the HA, NA, or M gene of the inhibitor-resistant Udorn virus on the inhibitor-sensitive PR8 virus backbone. Parental PR8 and Udorn viruses were also constructed from plasmids to serve as controls. An *in vitro* virus neutralization assay, developed to assess inhibition by saliva (11), was then performed on each of these viruses (Fig. 1A). At a dose of 5,000 PFU, Udorn virus was neutralized by mouse saliva relatively weakly (30% ± 5%), whereas PR8 virus was almost totally inhibited (93% ± 5%; *P* < 0.0001 compared to Udorn). Inhibition was also seen when either the M (95% ± 4%; *P* < 0.0001) or HA (86% ± 7%; *P* < 0.0001) gene of Udorn virus was expressed on the PR8 backbone, referred to as PR8(Ud-M) and PR8(Ud-HA), respectively. However, expression of the Udorn NA gene on a PR8 backbone in PR8(Ud-NA) virus resulted in a low level of neutralization comparable to that of parental Udorn virus (38% ± 2%; *P* > 0.05).

These data indicated that the viral NA was the critical determinant of sensitivity to the neutralizing inhibitor in mouse saliva. To support this, a hybrid Udorn virus bearing PR8 NA, referred to as Ud(PR8-NA) virus, was created. In the inhibition assay (Fig. 1A), this virus was associated with enhanced sensitivity to neutralization (72% ± 2%; *P* < 0.0001 compared to Udorn), although this was not as potent as that observed against the PR8 parent virus (*P* < 0.001 compared to PR8). Together, these data confirmed that the salivary inhibitor was indeed targeting the NA of PR8 virus to exert its effect.

We also tested the ability of these viruses to be neutralized following exposure to mouse saliva that had been treated with V. cholerae RDE to remove sialic acid residues (Fig. 1B). Confirming the results shown in the accompanying paper (11), the ability of mouse saliva to neutralize Udorn virus was markedly reduced by RDE, with only 16% ± 6% neutralization after treatment (compared to 30% ± 5% before) (Fig. 1A), but RDE-treated saliva retained virtually all its neutralizing activity against PR8 virus (87% ± 4% inhibition; *P* < 0.0001 compared to Udorn). Hybrid viruses containing PR8 NA, i.e., PR8(Ud-HA), PR8(Ud-M), and Ud(PR8-NA), were also sensitive to neutralization by RDE-treated saliva, while PR8(Ud-NA) virus containing the Udorn NA showed less inhibition once the sialic acid had been removed from the saliva (Fig. 1B).

### The NA of Udorn virus confers the ability to progress to the lungs after URT infection of mice.

**FIG 2:**
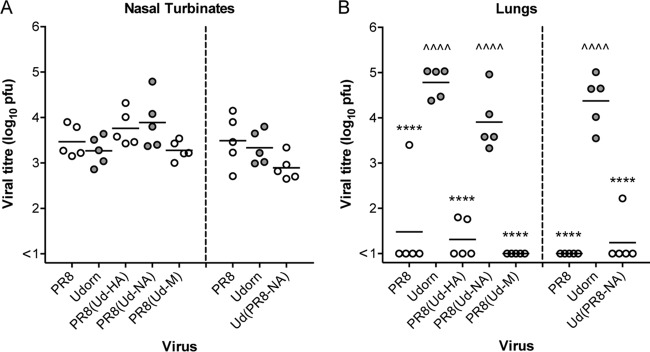
Viral titers in nasal turbinates and lungs of mice infected with reverse-engineered viruses via the URT route. BALB/c mice were infected with 10^4.5^ PFU of hybrid viruses on either a PR8 (left) or Udorn (right) virus backbone, together with reverse-engineered PR8 and Udorn virus as controls. After 6 days, nasal turbinates (A) and lungs (B) were removed, and viral titers in homogenates were determined by plaque assay on MDCK cell monolayers. Individual titers (circles) and the mean viral titers of 5 mice per group (horizontal lines) are shown, expressed as titers per organ. Filled circles, viruses containing Udorn NA; open circles, viruses containing PR8 NA. Compared to PR8, ^^^^, *P* < 0.0001. Compared to Udorn, ****, *P* < 0.0001.

Iida and Bang (9) first described the URT model of infection when they noted that an influenza virus infection could be restricted to the nasal epithelium of mice if the virus inoculum was administered to the external nares in a small volume without anesthetic. In the accompanying paper (11), we show that the RDE-resistant salivary inhibitor is responsible for preventing the progression of PR8 virus down the respiratory tree, while the RDE-sensitive inhibitor of Udorn virus did not have this capacity. Here, we investigated whether the presence of the PR8 NA was responsible for halting virus progression to the lungs. Groups of 5 BALB/c mice were inoculated intranasally (i.n.) as an URT infection with 10^4.5^ PFU of Udorn, PR8, or hybrid virus. Nasal turbinates (Fig. 2A) and lungs (Fig. 2B) were removed and assayed for the presence of infectious virus 6 days postinfection.

When hybrid viruses on a PR8 backbone were examined, high titers of virus were recovered from the nasal tissue of all the mice (Fig. 2A, left), indicating that expression of Udorn HA, NA, or M on a PR8 backbone did not affect the fitness of the viruses for replication in the upper respiratory tract (*P* > 0.05). In contrast, viral progression and subsequent replication in the lungs differed dramatically between these five viruses (Fig. 2B, left). While Udorn virus grew to high titers in the lungs of all the mice, PR8 virus was undetectable in 4/5 mice at this site (*P* < 0.0001 compared to Udorn). Of the hybrid viruses on a PR8 backbone, only PR8(Ud-NA) virus consistently progressed to the lungs and replicated to high titers (*P* > 0.05 compared to Udorn). PR8(Ud-M) virus was not detected in the lungs following URT inoculation, and PR8(Ud-HA) virus showed only low or undetectable titers (both *P* < 0.0001 compared to Udorn). When mice were infected with Ud(PR8-NA) virus, again, no statistically significant difference was observed in replication in the nasal turbinates compared to that of the parental viruses (Fig. 2A, right), yet the virus did not progress efficiently to the lungs (*P* < 0.0001 compared to Udorn), with virus detected at low titers in only 1/5 mice (Fig. 2B, right).

**FIG 3:**
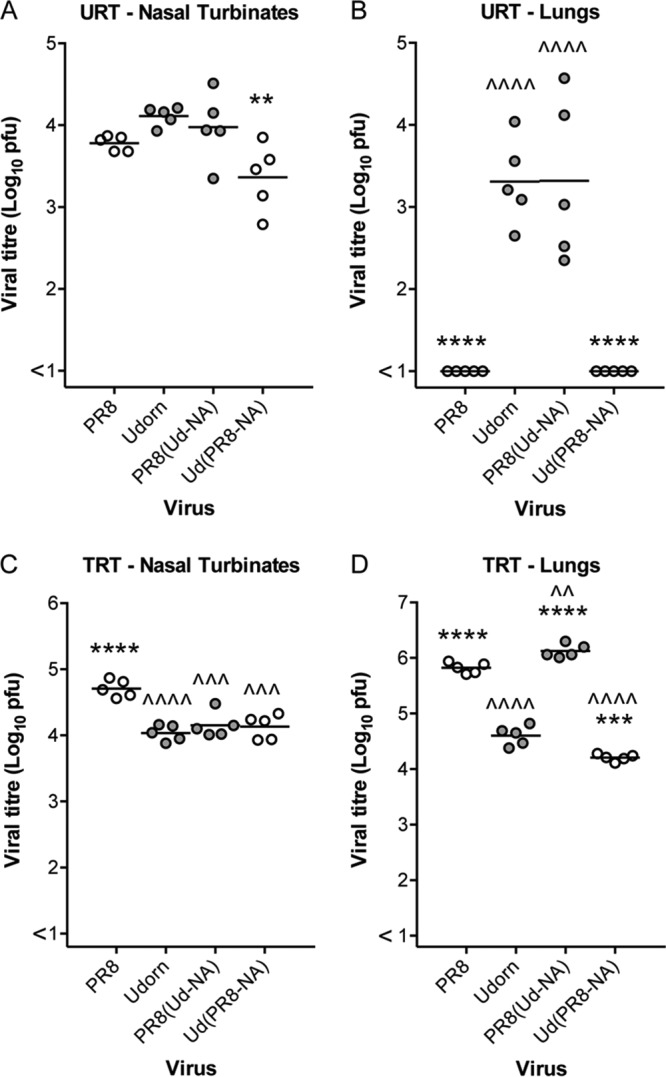
Viral titers in the nasal turbinates and lungs of mice infected with NA hybrid viruses. BALB/c mice were infected with 10^4.5^ PFU of NA hybrid and parent viruses via either the URT (A and B) or TRT (C and D) route. After 4 days, nasal turbinates (A and C) and lungs (B and D) were removed, and viral titers in homogenates were determined by plaque assay on MDCK cell monolayers. Individual titers (circles) and the mean viral titers of 5 mice per group (horizontal lines) are shown, expressed as titers per organ. Filled circles, viruses containing Udorn NA; open circles, viruses containing PR8 NA. Compared to PR8, ^^, *P* < 0.01; ^^^, *P* < 0.001; and ^^^^, *P* < 0.0001. Compared to Udorn, **, *P* < 0.01; ***, *P* < 0.001; and ****, *P* < 0.0001.

These differences were not due to an intrinsic inability of some strains that we had created to grow in the lungs. All grew to high titers when initially delivered to the total respiratory tract, as exemplified by direct comparison of URT (Fig. 3A and B) and TRT (Fig. 3C and D) delivery of Udorn, PR8, PR8(Ud-NA), and Ud(PR8-NA) viruses. Despite having mismatched HA and NA glycoproteins, the hybrid viruses replicated well in the lungs (Fig. 3D), in addition to the nasal turbinates (Fig. 3C), after TRT delivery, with PR8(Ud-NA) virus titers more closely resembling PR8 virus titers and Ud(PR8-NA) virus titers resembling Udorn virus titers. These data confirm that the inhibition of Ud(PR8-NA) virus, like that of PR8 virus itself, is achieved by stopping progression of virus infection to the lungs rather than blocking the capacity to replicate within the lung tissue.

### Salivary inhibitor reduces the enzymatic activity of NA.

**FIG 4:**
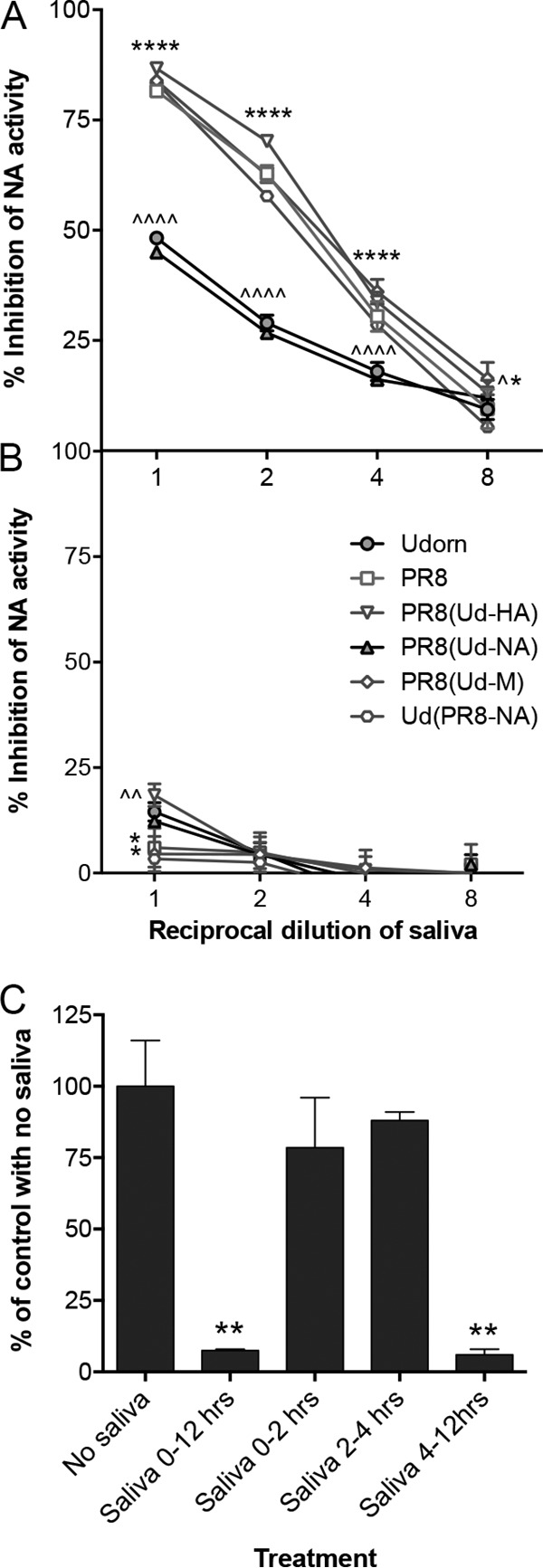
Inhibition of viral NA activity by untreated and RDE-treated saliva. (A and B) Standardized concentrations of reverse-engineered viruses were preincubated for 30 min at 37°C with serial 2-fold dilutions of either untreated (A) or RDE-treated (B) saliva, starting from a 9:1 (vol/vol) ratio of saliva to virus, followed by the addition of MUNANA substrate. The data show the percent inhibition of NA activity in the presence of saliva and are representative of the results of 2 individual experiments performed in duplicate. Filled symbols, viruses containing Udorn NA; open symbols, viruses containing PR8 NA. Compared to PR8, ^, *P* < 0.05 for PR8(Ud-M) at 1 in 8 dilution; ^^, *P* < 0.01 for PR8(Ud-HA) undiluted; and ^^^^, *P* < 0.0001 for Udorn and PR8(Ud-NA). Compared to Udorn, *, *P* < 0.05 for PR8(Ud-M) at 1 in 8 dilution (A), for PR8(Ud-M) undiluted (B), and for Ud(PR8-NA) undiluted (B); and ****, *P* < 0.0001 for PR8, PR8(Ud-HA), PR8(Ud-M), and Ud(PR8-NA). (C) PR8 virus was added to MDCK cell monolayers in TC96 wells at an MOI of 1 (10^5^ PFU), allowed to adsorb for 1 h at 37°C, and then removed by washing. Saliva at a 1 in 2 dilution was added and remained on the monolayers throughout the infection (0 to 12 h), was added and remained on the monolayers for the first 2 h followed by thorough washing and replacement with medium without saliva (0 to 2 h), was added after 2 h for an additional 2 h (2 to 4 h), or was added after 4 h for the remainder of the infection cycle (4 to 12 h). At 12 h postinfection, the supernatants were sampled, and the amount of infectious virus was determined by plaque formation in MDCK cells. The data are expressed as the percentages of virus from cultures containing no saliva, which had an infectious-virus yield of 10^6.47 ± 0.07^ PFU. The error bars represent the range of duplicate samples. **, *P* < 0.01 compared to no-saliva control.

Although the viral NA appeared to be the key determinant of susceptibility to the salivary inhibitor, it was unclear whether binding of the inhibitor to the NA also affected its sialidase function. To examine this, reverse-genetics-derived parent and hybrid viruses were preincubated with untreated or RDE-treated saliva, or with phosphate-buffered saline (PBS) as a control, and assayed for the ability to cleave the NA substrate 4-methyl-umbelliferyl-*N*-acetyl neuraminic acid (MUNANA) (Fig. 4). When preincubated with untreated saliva, the NA activities of viruses bearing the PR8 NA, i.e., PR8, PR8(Ud-HA), PR8(Ud-M), and Ud(PR8-NA) viruses, were strongly inhibited (Fig. 4A). In contrast, viruses bearing the Udorn NA, i.e., Udorn and PR8(Ud-NA) viruses, were markedly more resistant (Fig. 4A). Surprisingly, when viruses were preincubated with RDE-treated saliva, very little NA inhibition was observed for any strain tested (Fig. 4B). This was true despite the fact that RDE-treated saliva strongly inhibited viruses with a PR8 NA in the *in vitro* neutralization assay using the same saliva/virus ratio (Fig. 1). This suggested that saliva does not alter the intrinsic activity of the enzyme active site, but rather, the salivary inhibitor may block access to the site; for small substrates (e.g., MUNANA [mass, 489.4 Da]), inhibition of NA activity *in vitro* required sialylation of the salivary inhibitor, but for cleavage of larger substrates (e.g., sialic acid on cell surface glycoproteins and glycolipids), sialylation may not be required. Unfortunately, we found that saliva itself contained intrinsic sialidase activity that cleaved sialic acid from the larger NA substrate fetuin (48.4 kDa) independently of virus, precluding experiments to test the activity of the salivary inhibitor against larger substrates. The sialidase activity of saliva was not evident against the MUNANA substrate.

An alternative hypothesis, that the binding of the salivary inhibitor to NA sterically affects the binding of HA to its receptor, was ruled out in experiments examining the ability of saliva to prevent the hemagglutination of chicken red blood cells. Untreated saliva had very low levels of hemagglutination inhibition (HI) activity against Udorn, PR8, and hybrid viruses (titers ≤ 16), and this did not correlate with the inhibitor sensitivity of the tested viruses (data not shown). Furthermore, this weak HI activity was completely lost when the saliva was RDE treated, effectively ruling out HA as an additional viral target of the inhibitor.

To further investigate whether the inhibitor in saliva affects NA activity against large substrates, we took advantage of the fact that the sialidase function of NA prevents self-aggregation of newly formed virus particles and their reattachment to infected cells after budding, thereby aiding efficient virus release (12, 13). This implies that the inhibitory function of saliva for PR8 virus should be manifest predominantly when virus is exiting the cell after replicating. To examine this, we performed a time-of-addition assay in Madin-Darby canine kidney (MDCK) cells (Fig. 4C). Virus yields were decreased by at least 10-fold, or 90%, when saliva was present throughout the assay. No significant decrease was observed when the saliva was added for the initial 2 h after infection or from 2 to 4 h postinfection. In contrast, saliva added during the virus release stage (4 to 12 h) dramatically reduced the amount of virus recovered in the culture medium, consistent with a role for the inhibitor in blocking NA function.

**FIG 5:**
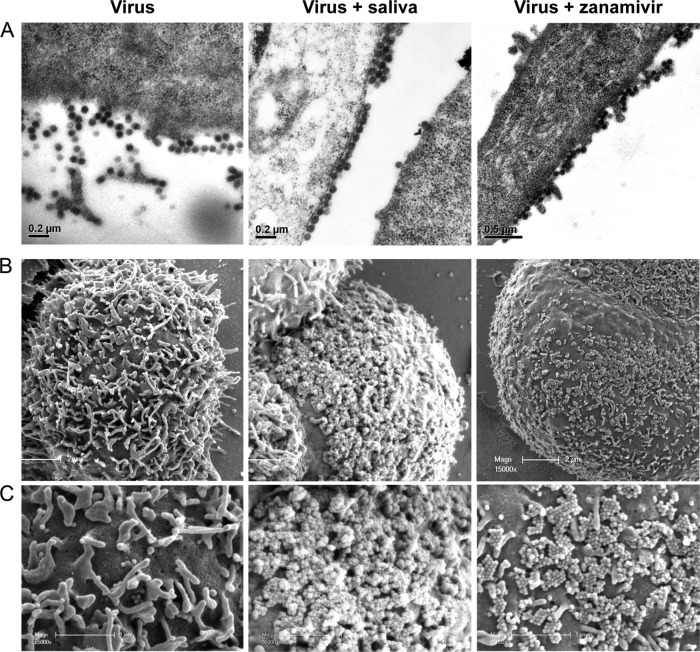
Electron microscopy of saliva-treated infected MDCK cells. (A) MDCK cells were exposed to PR8 virus (MOI = 10) in RPMI plus antibiotics for 1 h at 37°C and then washed thoroughly and cultured in the presence of medium, untreated saliva (1:2 final dilution), or 50 ng/ml of zanamivir for a further 10 h at 37°C. The cells were then fixed and embedded in resin. Ultrathin sections were stained and visualized by TEM. (B) Alternatively, MDCK cells were infected and seeded onto gold-coated coverslips. After 10 h of incubation as described above, the coverslips were fixed and visualized by SEM. (C) Higher-magnification (×65,000) images of the cell surfaces in panel B.

To support this conclusion, MDCK cells were infected with PR8 virus and cultured in the presence of medium alone, saliva, or zanamivir, the commercially available NA inhibitor drug, and then stained and visualized by transmission electron microscopy (TEM) (Fig. 5A) and scanning electron microscopy (SEM) (Fig. 5B and C). Following culture in medium alone, PR8 virus-infected cells displayed freely budding virus with only small numbers of virions fixed to the surface of the cell and little or no aggregated virus. In contrast, when PR8 virus-infected cells were cultured in the presence of saliva, considerably more virus was present at the budding surface, and it was visibly aggregated, suggestive of a failure to release from the cell efficiently. This effect was similar to that for infected cells in the presence of zanamivir.

**FIG 6:**
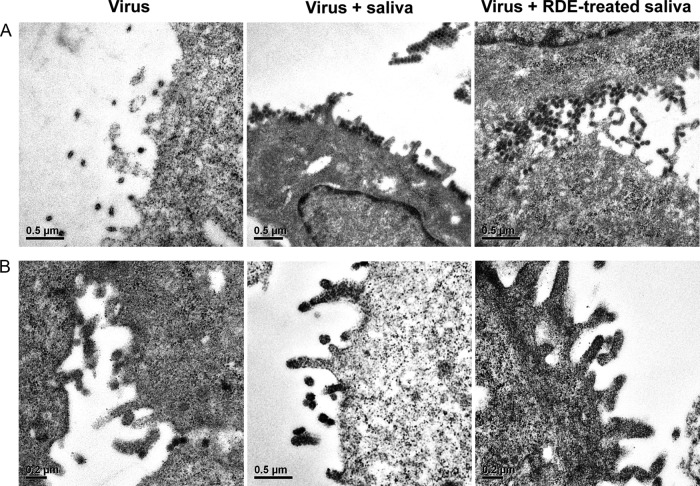
Electron microscopy of saliva-treated MDCK cells infected with Ud(PR8-NA) virus (A) or Udorn virus (B). The cells were infected as for Fig. 5A and cultured in the presence of medium or untreated or RDE-treated saliva (1:2 final dilution) for a further 10 h at 37°C. The cells were then fixed and embedded in resin. Ultrathin sections were stained and visualized by TEM.

Similar results were observed by TEM of cells infected with the Ud(PR8-NA) virus (Fig. 6A). In the presence of saliva, virus accumulated at the surface of the cell and could be observed in aggregates. This was in direct contrast to results with the Udorn parent virus, which showed no virus accumulation at the cell surface in either the presence or absence of saliva (Fig. 6B), suggesting that budding virus could be freely released into the surrounding medium. Cell surface-bound virus was also present when the Ud(PR8-NA) virus-infected cells were incubated in the presence of RDE-treated saliva (Fig. 6A), consistent with our previous observation that the inhibitory function was not sialic acid dependent. These data indicated that the salivary inhibitor did indeed compromise the function of the enzyme active site of the viral NA, as does zanamivir, but the sialic acid independence suggests the mechanism may be somewhat different from the sialic acid analog, which binds tightly in the active-site pocket.

### Sensitivity to the salivary inhibitor of a panel of H3N2 hybrid viruses also correlates with the NA.

**FIG 7:**
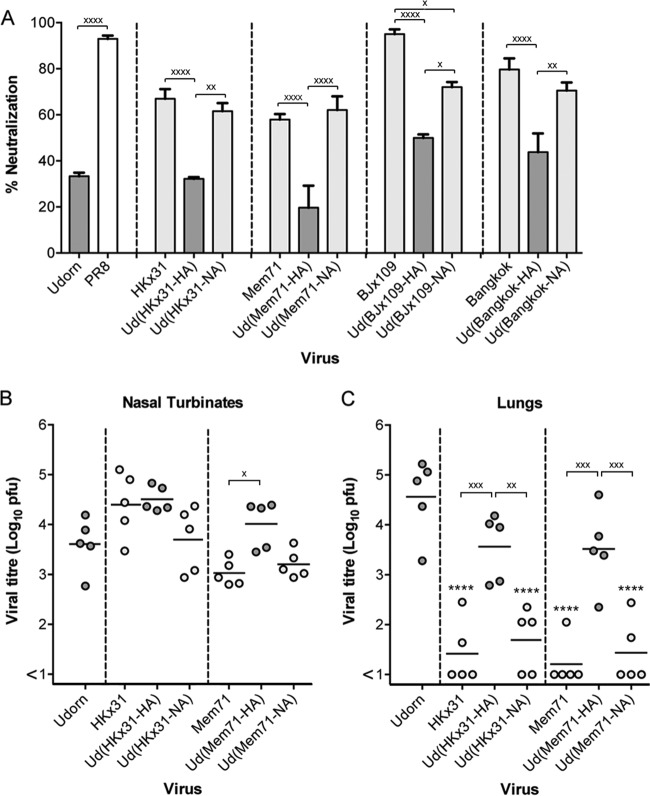
*In vitro* neutralization and *in vivo* progression of H3N2 hybrid viruses. Viruses (5,000 PFU) were mixed with untreated saliva or RPMI plus BSA as a control at a 9:1 (vol/vol) ratio of virus to saliva. The mixtures were then incubated at 37°C for 30 min before being added to MDCK cell monolayers, and viral infectivity was determined by plaque formation. The percent neutralization of each viral strain by saliva is shown (A). The viral origin of the NA is denoted by differently shaded bars: Udorn, dark gray; donor H3N2, light gray; and PR8, white. The data represent means and standard deviations of the results of at least 2 individual experiments, each performed in triplicate. BALB/c mice were infected via the URT with 10^4.5^ PFU of the indicated hybrid viruses. After 6 days, nasal turbinates (B) and lungs (C) were removed, and viral titers in homogenates were determined by plaque assay on MDCK cell monolayers. Individual titers (circles) and the mean viral titers of 5 mice per group (horizontal lines) are shown, expressed as titers per organ. Dark-gray circles, viruses containing Udorn NA; light-gray circles, viruses containing a donor H3N2 NA. For the indicated comparisons: x, *P* < 0.05; xx, *P* < 0.01; xxx, *P* < 0.001; xxxx, *P* < 0.0001. Additionally, in panel A, all dark-grey bars are significantly different from PR8 (*P* < 0.0001). In panel C, ****, *P* < 0.0001 compared to Udorn.

To further investigate the target of the inhibitor and to define the precise residues involved, we created a panel of hybrid viruses by engineering the HA or NA genes of other closely related H3N2 strains with differing sensitivities to the salivary inhibitor on a Udorn backbone. The susceptibilities of Udorn viruses expressing either the HA or NA of HKx31, A/Memphis/1/71 (Mem71), BJx109, or A/Bangkok/1/79 (BK79) donor virus were tested in the saliva neutralization assay (Fig. 7A) and compared to those of the parent viruses from which these genes were derived and to that of Udorn virus. This approach was undertaken with a view to aligning the NA sequences of these closely related strains to identify amino acid differences that could potentially define resistant versus susceptible strains. Of the 4 sensitive parental donor strains chosen, HKx31 and Mem71 were markedly less susceptible to the salivary inhibitor than BJx109 and BK79 (66.9% ± 7.3% and 58.0% ± 3.3% versus 95.1% ± 2.9% and 79.7% ± 8.3%, respectively). The relative susceptibilities of a wider panel of H3N2 strains reported in the accompanying paper (11) are summarized in Table 1. When Udorn hybrid viruses expressed the HAs of the donor strains, their neutralization by saliva remained low and comparable to that of the Udorn virus (Fig. 7A). However, when Udorn virus expressed the NAs of the donor strains, neutralization by saliva was significantly increased. With the exception of the hybrid virus bearing BJx109 NA, susceptibility was equivalent to that of the respective donor strain (*P* > 0.05). Neutralization of the Ud(BJx109-NA) virus was statistically significantly higher than that of Ud(BJx109-HA) (*P* < 0.05) despite not reaching the level of the donor virus.

**TABLE 1:** Susceptibilities of influenza virus strains to the salivary inhibitor

Virus strain	% homology to A/Udorn/307/72 virus (N2)	Degree of susceptibility^*a*^	Amino acid sequence^*b*^ (residues 359–381 of NA)
Consensus			DVWMGRTIS D RSGYETFKVIG
A/Northern Territory/60/68 (N2)	94.0	++++	.........K.L...........
A/Philippines/2/68 (N2)	94.5	+++	.........K.L...........
A/Aichi/2/68 (N2)	94.0	+++	.........K.L...........
A/England/878/69 (N2)	96.8	++++	.........K.L...........
A/Memphis/1/71 (N2)	97.7	++++	.........K.S...........
A/Udorn/307/72 (N2)	100	+	.........E.S...........
A/Port Chalmers/1/73 (N2)	99.4	+	.........E.S...........
A/Victoria/3/75 (N2)	98.7	+	.........E.S...........
A/Bangkok/1/79 (N2)	97.0	+++++	.........EES...........
A/Beijing/353/89 (N2)	93.8	+++++	........GEEL...........
A/Puerto Rico/8/34 (N1)	42.1	+++++	G..I...K.HSS.H.F.MIWDPN

a Susceptibility to saliva determined by *in vitro* virus neutralization assay. The ratings indicate the degree of susceptibility (+, <40%; +++, 50 to 69%; ++++, 70 to 89%; +++++, >90% neutralization by saliva; summarized from Ivinson et al. (11).

b Amino acid sequences of residues 359 to 381 of NA (N2 numbering) with differences from the consensus sequence indicated.

To determine whether the *in vitro* susceptibility and resistance of these viruses predicted their ability to progress to the lungs *in vivo*, mice were inoculated by the URT route, and virus titers were determined in the nose and the lungs at day 6 postinfection. Despite growing to high titers in the nasal turbinates (Fig. 7B), progression of HKx31 virus to the lungs was less efficient than that of Udorn virus (Fig. 7C) (*P* < 0.0001). This was also apparent for mice infected with the Udorn virus expressing the NA of HKx31 (*P* < 0.0001). Conversely, both the Udorn strain and Ud(HKx31-HA) virus consistently traveled to the lungs and grew to high titers in all the mice examined (*P* > 0.05). Viruses with Mem71 HA and NA on a Udorn backbone showed the same pattern of tropism as the HKx31 hybrids (Fig. 7C). Similar experiments could not be performed for BJx109 and BK79 viruses, as these strains do not establish a productive infection in mouse lungs (8) due to their susceptibility to the innate inhibitor SP-D (14).

### Amino acids 368 to 370 of NA determine susceptibility to the inhibitor.

**FIG 8:**
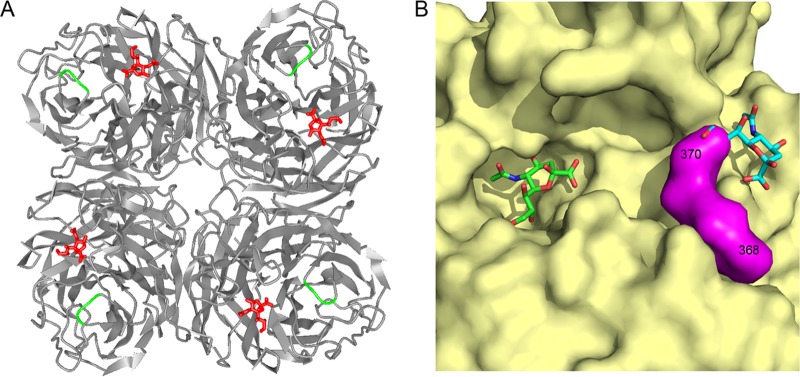
NA residues 368 to 370 are located on the surface of the NA, close to the rim of the enzymatic site. (A) Ribbon structure of the N2 NA molecule showing the interaction and positioning of the sialic acid moiety (twist-boat conformation) in red in the catalytic site. Residues 368 to 370 are shown in green. Protein Data Bank (PDB) code, 2BAT. The figure was generated in iCn3D. (B) Surface depiction of the structure of one subunit of an N2 (A/Tokyo/3/67) neuraminidase, colored yellow apart from the loop segment 368 to 370, which is shown in magenta. In A/Tokyo/3/67, the sequence is K368 D369 L370, and the side chains of all three residues are exposed on the surface. Sialic acid in the active site is depicted in green. PDB code, 2BAT. The cyan-colored sialic acid is derived from an overlay with an avian N9 neuraminidase that has a second site for binding, but not cleaving, sialic acid. PDB code, 1MWE. The figure was generated in PyMOL.

To determine the precise binding site of the salivary inhibitor, the amino acid sequences of the NAs from the virus panel were compared. Despite possessing differing degrees of sensitivity to saliva, the NAs of these closely related strains shared a high degree of homology to Udorn NA, ranging from 93.8% to 99.4% (Table 1). This is in contrast to the amino acid sequences of PR8 (N1) and Udorn (N2) NAs, which, belonging to different subtypes, shared only low homology (42.1%). We focused on a region of the N2 NA spanning amino acid residues 368 to 370, as this was the only location that uniquely differentiated the H3N2 viruses according to salivary inhibitor susceptibility. Udorn, A/Port Chalmers/1/73, and A/Victoria/3/75 viruses all had an EDS sequence at positions 368 to 370 of the NA, whereas other N2 viruses (in order of increasing susceptibility) had KDL, KDS, EEL, and EES. The N1 NA from the highly susceptible PR8 virus had HSS at the corresponding locations (residues 350 to 352 in N1 numbering). The 368-to-370 region of the N2 immediately precedes the conserved 371R, which interacts with the carboxylate group of the sialic acid substrate (15) and is flanked on either side by highly conserved regions within the N2 subtype. Figure 8 shows the location of this region on the 3-dimensional crystal structure of the N2 NA. The three residues are situated on the surface of NA adjacent to the active site, potentially making this region readily accessible for recognition by the salivary inhibitor.

**FIG 9:**
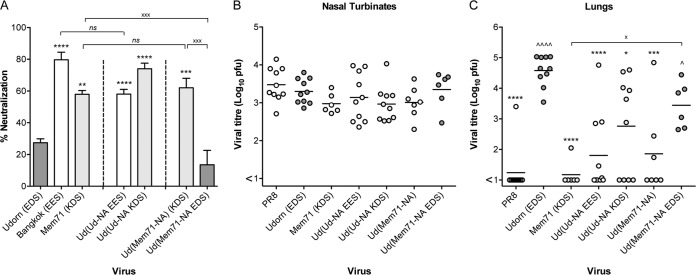
*In vitro* neutralization and *in vivo* progression of mutant Udorn viruses. (A) Udorn viruses (5,000 PFU) bearing changes in amino acid residues 368 to 370 of the viral NA were mixed with untreated saliva or RPMI plus BSA as a control at a 9:1 (vol/vol) ratio of virus to saliva. The mixtures were then incubated at 37°C for 30 min before being added to MDCK cell monolayers, and viral infectivity was determined by plaque assay. The percentages of virus neutralized by saliva are shown. The amino acid sequence from 368 to 370 is denoted by differently shaded bars: EDS, dark gray; KDS, light gray; EES, white. The data represent means and standard deviations of the results of at least 2 individual experiments, each performed in triplicate. (B and C) BALB/c mice were infected via the URT route with 10^4.5^ PFU of each virus. After 6 days, nasal turbinates (B) and lungs (C) were removed, and viral titers in homogenates were determined by plaque assay on MDCK cell monolayers. Individual titers (circles) and the mean viral titers of 6 to 10 mice per group (horizontal lines) are shown, expressed as titers per organ. The residues in parentheses denote the unmodified or genetically altered sequence. Dark-gray circles, viruses containing NA bearing EDS; light-gray circles, viruses bearing KDS; open circles, viruses bearing EES. The PR8 NA sequence is HSS at the corresponding location. Compared to Udorn, *, *P* < 0.05; **, *P* < 0.01; ***, *P* < 0.001; and ****, *P* < 0.0001. Compared to PR8, ^, *P* < 0.05; and ^^^^, *P* < 0.0001. For other important comparisons indicated by a bracket, x, *P* < 0.05; xxx, *P* < 0.001; and ns, not significantly different.

To determine whether residues 368 to 370 were involved in binding the salivary inhibitor, we generated Udorn viruses containing D369E or E368K mutations in the viral NA, changing the consensus sequence from the putative resistance motif EDS to EES (naturally occurring in BK79) or KDS (in Mem71), respectively, which are found in inhibitor-sensitive strains. When tested for inhibition by mouse saliva in the *in vitro* virus neutralization assay (Fig. 9A), introduction of the D369E (EES) mutation was found to significantly increase neutralization by saliva of the resistant Udorn (EDS) virus from 27.4% ± 9.4% to 58.1% ± 6.7% (*P* < 0.0001). Similarly, introduction of the E368K (KDS) mutation into Udorn NA increased neutralization of the resulting virus to 74.0% ± 7.1% (*P* < 0.0001).

To support these findings, Udorn virus was engineered to contain the NA of Mem71 in which the introduction of a K368E mutation changed the KDS motif to EDS of the resistant Udorn strain. When tested for neutralization by saliva *in vitro*, Udorn(Mem71-NA) showed 62.1% ± 8.4% neutralization, similar to Mem71 virus (58.0% ± 3.3%; *P* > 0.05). However, when Udorn virus expressed the NA of Mem71 bearing an EDS motif instead of KDS, the virus was now largely resistant to the salivary inhibitor (13.6% ± 12.8% neutralized; *P* > 0.05 compared to Udorn), confirming the importance of residues 368 to 370 in determining *in vitro* sensitivity to the salivary inhibitor.

### EES and KDS mutations at residues 368 to 370 of NA limit progression of Udorn virus to the lungs of mice.

To determine the effect of mutations in the *in vitro* susceptibility motif at N2 residues 368 to 370 on progression of the virus down the respiratory tract, groups of 6 to 10 mice were inoculated via the URT route with 10^4.5^ PFU of the reverse-engineered mutant and parent viruses, and titers in the nose (Fig. 9B) and lungs (Fig. 9C) were examined 6 days after infection. All the viruses tested grew to similar levels in the nasal turbinates, indicative of comparable replicative fitness (Fig. 9B). The PR8 and Udorn viruses used as controls showed the expected lung tropism (Fig. 9C), with PR8 virus infrequently detected in the lungs at day 6 (1 of 10 mice) and Udorn virus progressing to the lungs and replicating to high titers in all 10 mice. Introduction of the EES motif into the Udorn NA had a significant impact on the ability of the virus to consistently travel to the lungs, with undetectable virus or very low titers in 70% (7/10) of the mice and a mean viral titer more than 300-fold lower in the lungs than in those from mice infected with unmodified Udorn virus (*P* < 0.0001). Introduction of the KDS motif into the Udorn NA also reduced the ability of the virus to progress to the lungs, but the effect was not as pronounced as the change to EES, showing 40% (4/10) of the mice with undetectable pulmonary virus and a 30-fold reduction in the mean viral titer compared to unmodified Udorn virus (*P* < 0.05). In addition, the progression of Mem71 virus to the lungs showed the virus to be highly impeded (Fig. 9C), consistent with the data in Fig. 7C, and the hybrid virus consisting of Mem71 NA on a Udorn virus backbone progressed to the lungs in only 3 of 7 mice, with approximately 300-fold lower mean virus titer than for Udorn virus (*P* < 0.001). However, introduction of the EDS motif in Ud(Mem71-NA EDS) virus resulted in the virus progressing to the lungs in 100% of the mice (6/6). These data show that, despite breakthrough pulmonary replication in some individual animals, assessment of the overall ability of engineered viruses to be halted in their progression to the lungs is a reflection of their capacity to be neutralized by the salivary inhibitor *in vitro*.

## DISCUSSION

This work identified a site on influenza virus neuraminidase that confers sensitivity to a previously undescribed natural inhibitor of the virus in mouse saliva, and to our knowledge, this is the first description of any innate inhibitor of influenza virus specifically targeting this viral protein. This is significant in providing insight into a novel antiviral defense mechanism that may have evolved to inhibit the spread of virus from the upper respiratory tract to protect the trachea and lungs. Although laboratory mice are routinely used to study influenza virus pathogenesis, as far as we know, they do not naturally succumb to influenza in the wild. Our work shows that mice already produce their own natural form of NA inhibitor, with a function similar to that which humans have produced synthetically in the form of zanamivir and oseltamivir to reduce the burden of influenza virus infection. This would render virus naturally transmitted to the URT inefficient in causing pulmonary infection in these animals, despite the virus being able to grow to high titers in the lungs if delivered directly to that site, as when infected experimentally. Whether similar inhibitors are also naturally produced in other species is not known. We were unable to demonstrate an inhibitor with equivalent virus strain specificity in ferret saliva and saliva taken from human infants before any exposure to influenza virus (unpublished findings).

The presence of inhibitors in saliva would appear to be a useful protective mechanism against respiratory pathogens, and there have been various reports of inhibition of influenza virus (4, 16) and other viruses (17, 18) by human saliva. White et al. (16) showed that among purified human salivary proteins, MUC5B, scavenger receptor cysteine-rich glycoprotein 340 (salivary gp340), histatins, and human neutrophil defensins inhibited IAV at concentrations present in whole saliva. However, both salivary gp340 and MUC5B inhibited infectivity and HA activity of IAV by presenting sialic acid ligands that bound to the viral HA, preventing attachment to target cells. Hartshorn et al. (4) have also described the antiviral effects of salivary gp340 isolated from human donors that were mediated by calcium-independent interactions between the virus and sialic acid-bearing carbohydrates on gp340. The inhibitor described here is clearly different from these in its mode of action, and further studies to determine its identity would be of interest due to its novelty.

The activity of the inhibitor against N2 virus strains was shown to be critically dependent on amino acid residues 368 to 370 of the NA, so that NAs bearing EDS at these positions were resistant and those with EES/EEL/KDS were sensitive. Although change of an aspartic acid (D) residue at position 369 to a glutamic acid (E) residue (EDS to EES) was the most conservative change, it had the most pronounced effect, increasing susceptibility to saliva *in vitro* but also significantly reducing the ability of the Udorn EES virus to progress to the lungs after URT infection compared to the wild-type strain. Although D and E residues have similar properties and substitution of these residues maintains the same overall charge, glutamic acid has a longer side group than aspartic acid, potentially altering binding of the salivary inhibitor. The other amino acid substitutions resulted in changes to charge, polarity, hydrophobicity, or acidity, which may also affect potential binding.

Despite the similarity of functional inhibition as visualized by electron microscopy (EM), the mechanism of action of the salivary inhibitor appears to be different from that of the synthetic NA inhibitor antiviral drugs. These compounds are sialic acid analogs that function by tightly binding to the NA active site, blocking interaction with receptor-bound sialic acid (19). This inhibits the natural catalytic activity of sialic acid on the cell surface during budding of the virus, thereby hindering release of newly formed virions from infected cells and restricting spread of the virus (20). The ability of the salivary inhibitor to neutralize virus infectivity for MDCK cells *in vitro* and to cause aggregation of virus at the budding sites of infected cells, even after RDE treatment, indicates that the action of the inhibitor is sialic acid independent and therefore unlikely to act by binding within the active site. We therefore propose that the salivary inhibitor binds near the active site and occludes entry of the viral receptor in a similar way to neuraminidase-inhibiting (NI) antibody. Residues 368 to 370 are located on the surface of the NA near the outer rim of the active site, making them ideal candidates to be involved in such binding (Fig. 8A and B). The ability of saliva to inhibit cleavage of the small substrate MUNANA, but not when the saliva was RDE treated, may seem at odds with this proposal. However, this finding suggests that the NA-bound inhibitor is of sufficient size to inhibit the NA active site from engaging with both the viral receptor and the MUNANA substrate; when sialic acid is removed from the inhibitor by RDE, it can still bind to NA, but occlusion of the active site may be only partial. Access to the viral receptor may be prevented; however, the much smaller MUNANA can still gain access. This is similar to the observation that neuraminidase-inhibiting antibodies that block the ability of virus to cleave sialic acid from large substrates also have difficulty blocking access to MUNANA (J. McKimm-Breschkin, unpublished data). To formally address this hypothesis, larger sialic acid-containing substrates, such as fetuin, need to be tested. However, we found that saliva exhibited intrinsic sialidase activity against fetuin, precluding its use.

It was noted that swapping of residues 368 to 370 from the NA of inhibitor-sensitive virus strains onto a Udorn backbone did not result in complete inhibition of progression of virus to the lungs of all individual mice given a URT infection. It may be that to help facilitate binding of the salivary inhibitor to the surface of the NA, additional residues, in conjunction with 368 to 370, are involved. We investigated a number of candidate residues both within the active site (Q220K and I290V) and distal to the site (H155Y and N328K) that may have either affected the interaction of highly conserved catalytic residues with sialic acid (D151, R152, R224, E227, and R292) (15, 21, 22) or aided binding to the surface of the NA. All of these residues differed between Udorn NA and those of the highly susceptible Beijing and Bangkok viruses, yet none of these substitutions significantly enhanced inhibition (data not shown).

It is interesting that residues 368 to 370 form one of three loop structures that in avian NAs form a secondary sialic acid binding site (Fig. 8B). Initially discovered through red blood cell binding activity exhibited by purified N9 NA (23), this hemabsorbing (HB) site was eventually localized to residues on the outer rim of the NA binding pocket that played no role in NA catalytic activity (24). Varghese et al. (25) provided structural proof of the existence of this secondary sialic acid binding site using X-ray crystallography. Although not a function of NAs of human influenza viruses, this HB activity was shown to be transferable to the N2 of A/Tokyo/3/67 by site-directed mutagenesis, replacing amino acids in loops 368 to 370 and 399 to 403 with those from an avian N9 NA (26). Despite the importance of residues 368 to 370 in binding of the salivary inhibitor, we have no evidence that the same HB binding pocket is utilized by the inhibitor. What this second binding site does demonstrate, however, is that these loop structures outside the enzyme active site also form a site of interaction for other molecules, which in this case may have been exploited by the murine innate inhibitor system.

## MATERIALS AND METHODS

### Cells and viruses.

MDCK cells and 293T human embryonic kidney (293T) cells were maintained in RPMI 1640 (Sigma-Aldrich, Castle Hill, New South Wales, Australia) and Dulbecco's modified Eagle's medium (DMEM) (Life Technologies, Mulgrave, Victoria, Australia), respectively, supplemented with 10% heat-inactivated fetal calf serum (FCS) (Life Technologies), 2 mM l-glutamine (Sigma-Aldrich), 2 mM sodium pyruvate (Thermo Fisher, Scoresby, Victoria, Australia), 24 μg/ml gentamicin (Pfizer, West Ryde, New South Wales, Australia), 50 μg/ml streptomycin (Life Technologies), and 50 IU/ml penicillin (Life Technologies). Medium as described above, without serum, was used for dilutions, and the same medium containing 1 mg/ml bovine serum albumin (BSA) (RPMI plus BSA) was used as a control for virus neutralization assays. All media were sterilized by 0.45-μm-pore-size membrane filtration and stored at 4°C. All the cells were maintained at 37°C in 5% CO_2_.

Influenza viruses were propagated in 10-day-old embryonated hen's eggs at 35°C for 2 days before allantoic fluid was harvested and stored at −80°C. HKx31 (H3N2) influenza virus is a laboratory-derived reassortant consisting of the internal proteins of PR8 (H1N1) with the surface proteins of A/Aichi/2/68 (H3N2) (27, 28). BJx109 (H3N2) virus is a laboratory-derived reassortant consisting of the internal proteins of PR8 with the surface proteins of A/Beijing/353/89 (H3N2) (29). A/Memphis/1/71 (Mem71), and A/Bangkok/1/79 (BK79), both H3N2 viruses, were also used in this study. All the other influenza A viruses were generated using an eight-plasmid reverse-genetics system described previously (30). These included PR8 and Udorn (H3N2) viruses created as controls, and viruses consisting of either the PR8 backbone with the HA, NA, or M gene from Udorn virus or the Udorn backbone with the NA gene of PR8 virus. Udorn viruses bearing the HA or NA gene from either HKx31, Mem71, BJx109, or BK79 were also made, as well as mutant Udorn viruses containing either E368K (KDS) or D369E (EES) mutations in the viral NA and a reverse-engineered Udorn virus containing the NA gene from Mem71 virus bearing a K368E (EDS) mutation.

### Plasmid construction.

Total RNA was isolated from virus-infected allantoic fluid using an RNeasy kit (Qiagen) according to the manufacturer's instructions. The cDNA of PR8 and Udorn viruses was synthesized by reverse transcription of viral RNA (vRNA) with an oligonucleotide (Uni12 primer) complementary to the conserved 3′ end of the vRNA. The cDNA was amplified by PCR with segment-specific primers containing a BsmBI, BsaI, or AarI restriction site (31), and the products were cloned into the pHW2000 expression vector (30). The primer sequences are available upon request. All the clones were confirmed by full-length sequencing.

Mutations were introduced into the Udorn NA gene using custom primers designed to incorporate the desired amino acid substitutions, E368K (EDS to KDS) and D369E (EDS to EES). Primers were also used to incorporate a K368E (KDS-to-EDS) mutation into the Memphis NA. Two complementary products were amplified by PCR and then used as templates in another round of PCR with the universal primers to generate the full-length NA gene encoding the mutations. The custom primer sequences are available upon request.

### Transfection and rescue of viruses.

An 8-plasmid reverse-genetics system described previously (30) was used to create influenza viruses of the required genotypes. The day before transfection, confluent 293T and MDCK cells were trypsinized, and 2 × 10^6^ cells of each were added to 36 ml of Opti-MEM (Life Technologies) without FCS, supplemented with penicillin and streptomycin (termed Easy Flu medium). Each well of a 6-well tissue culture (TC6) plate was then seeded with 3 ml of this cell suspension (1 × 10^5^ to 3 × 10^5^ cells/well). FuGene6 transfection reagent (Promega, Alexandria, NSW, Australia) was used to transfect the cells according to the manufacturer's instructions. Briefly, 2 μl of FuGene6/μg of each plasmid was mixed in Opti-MEM, incubated at room temperature for 45 min, and added to subconfluent 293T and MDCK cell cocultures. The transfection medium was carefully removed after 6 h of incubation at 37°C and replaced with 1 ml of Easy Flu medium for a further 24 h. Another 1 ml of Easy Flu medium containing 2 μg of l-(tosylamido-2-phenyl) ethyl chloromethyl ketone (TPCK)-trypsin was then added to each well. The cells were incubated at 37°C until 72 h posttransfection, when cytopathic effects (CPE) were observed. Supernatants were then harvested and clarified by centrifugation, and rescued virus was amplified in 10-day-old embryonated hens' eggs. The identities of the reverse-engineered viruses were confirmed by restriction digestion and full-length sequencing of RT-PCR products for the HA, NP, NA, M (encoding the surface ion channel protein M2, in addition to the matrix protein), and NS genes.

### Mice and infection.

Inbred 6- to 8-week-old BALB/c mice (H-2^d^) were bred in the Animal Facility of the Department of Microbiology and Immunology, University of Melbourne, under specific-pathogen-free conditions and used with the approval of the University of Melbourne Animal Ethics Committee.

To establish a URT infection, mice that were not anesthetized were inoculated i.n. with 10^4.5^ PFU of PR8, Udorn, or reverse-engineered virus in 10 μl of PBS. To establish a TRT infection, mice were lightly anesthetized by isoflurane inhalation, and 50 μl of virus was delivered to the external nares to be breathed in smoothly by the animal. After 4 and 6 days, supernatants from nasal turbinate and lung homogenates were prepared, and viral titers (PFU per organ) were determined by plaque assay on MDCK cell monolayers (32).

### Collection of saliva.

Mice were anesthetized using isoflurane and injected intraperitoneally (i.p.) with 200 μl of 20 μg/ml Carbachol (carbamycholine chloride; Sigma, St. Louis, MO, USA) in PBS, and saliva was collected. Where specified, the saliva was treated with RDE. One volume of saliva in 8 volumes of Ca-Mg saline (0.24 mM CaCl_2_, 0.8 mM MgCl_2_, 20 mM boric acid, 0.14 mM sodium tetraborate, 0.15 M NaCl) was treated with 1 volume of V. cholerae RDE (Sigma, St. Louis, MO, USA) for 30 min at 37°C. The final concentration of RDE was 50 mU/ml. The RDE was then inactivated by incubation at 56°C for 1 h. Samples were transferred to 1,000-MW-cutoff Spectrapor membrane tubing (Pacific Laboratory Products, Victoria, Australia) and dialyzed against 50 mM NH_4_HCO_3_ at 4°C overnight. Following freeze-drying, samples were reconstituted to their original volumes with PBS and stored at −20°C. Control saliva was untreated, as we had previously shown that the inhibitor is resistant to heat treatment and lyophilization (11).

### Virus neutralization assay.

Plaque reduction on MDCK cell monolayers cultured in TC6 plates was used to measure neutralization of virus infectivity. Dilutions of virus were prepared in RPMI medium to give approximately 5,000 PFU per 10 μl and then mixed with untreated or RDE-treated saliva at a 1:9 (vol/vol) virus/saliva ratio. Mixtures of virus with RPMI plus BSA (1 mg/ml) at the same ratio were used as a control. The mixtures were then incubated at 37°C for 30 min before being added to MDCK cells, which were then overlaid, and infectious virus was enumerated by the ability to form plaques as described previously (32). Virus neutralization was expressed as the percent reduction in titer of virus obtained by preincubation with mouse saliva compared to that recovered after incubation with the control.

### Preparation of cells for EM.

MDCK cells were exposed to PR8 or Udorn virus at a multiplicity of infection (MOI) of 10 for 1 h at 37°C. The cells were then washed thoroughly and cultured in the presence of untreated or RDE-treated saliva (1:2 final dilution), medium alone, or 50 ng/ml of zanamivir for a further 10 h at 37°C.

For TEM analysis, cells were pelleted and resuspended in 2.5% glutaraldehyde in PBS for 2 h at room temperature before postfixing in 1% osmium tetroxide in buffer for 1 h. The cells were rinsed three times in fresh buffer for 10 min between treatments. The cells were then dehydrated in increasing concentrations of 10%, 30%, 50%, 70%, 90%, and two changes of 100% anhydrous ethanol for 15 min each. Following dehydration, the cells were infiltrated with increasing concentrations of 25%, 50%, 75%, and 100% LR white resin (ProSciTech, Thuringowa, Queensland, Australia) in ethanol for 6 h each. After a second change of 100% resin, the cells were embedded in fresh resin in gelatin capsules and allowed to gently sink to the bottom to form a loose pellet. The gelatin capsules were capped to exclude air, and the resin was polymerized in an oven at 60°C for 24 h. Embedded cells in blocks were sectioned with a diamond knife on a Leica Ultracut R microtome, and ultrathin (90-nm) sections were collected onto pioloform-coated 100-mesh hexagonal copper grids. The sections on grids were sequentially stained with saturated uranyl acetate for 10 min and Triple Lead Stain for 5 min (33) and viewed in a Phillips CM120 Biotwin transmission electron microscope at 120 kV.

For SEM analysis, 13- mm coverslips (Nunc) were gold coated in an Edwards S150B sputter coater (Edwards, West Sussex, England) for 5 min, giving a coating of approximately 30- to 40-nm gold thickness. MDCK cells treated as described above were placed onto the coverslips in 24-well plates and incubated at 37°C and 5% CO_2_ for 10 h.

The coverslips were removed and dehydrated in increasing concentrations of ethanol as for TEM (34). The coverslips were dried in a Bal-Tec CPD 030 critical-point dryer (Balzers, Liechtenstein, Germany) and mounted onto 25-mm aluminum stubs with double-sided carbon tabs. They were then coated with 10 nm of chromium using a Xenosput sputter coater (Dynavac, Wantirna South, Australia). The cells on coverslips were imaged with a Philips XL30 field emission scanning electron microscope (Philips, Eindhoven, Netherlands) at a voltage of 2.0 kV and a spot size of 2.

### Inhibition of NA activity.

NA activity was measured using the MUNANA fluorescence-based assay as described previously (35). The assay measures the increase in fluorescence of free methylumbelliferone released from MUNANA after cleavage by the neuraminidase. Viruses, standardized to 100 fluorescence units, were preincubated in microtiter plates for 30 min at 37°C with serial 2-fold dilutions of either untreated or RDE-treated saliva, starting from a 9:1 (vol/vol) ratio of saliva to virus. MUNANA (Carbosynth, Compton, United Kingdom) was then added, the assay mixtures were incubated for 1 h at 37°C, and the assays were stopped by the addition of 0.2 M Na_2_CO_3_. The plates were read in a PerkinElmer fluorimeter with an excitation wavelength of 365 nm and an emission wavelength of 450 nm.

### Hemagglutination inhibition assay.

Tests were performed in round-bottom 96-well microtiter plates at room temperature using 1% (vol/vol) chicken erythrocytes. Hemagglutination titers were determined by titration of virus samples in PBS, followed by addition of an equivalent volume of chicken erythrocytes. For hemagglutination inhibition tests, dilutions of untreated or RDE-treated saliva were prepared in PBS, and 4 hemagglutinating units (HAU) of virus was added. Following 30 min of incubation, chicken erythrocytes were added, and the ability of the saliva to inhibit virus-induced hemagglutination was assessed after 30 min.

### Statistical analysis.

All statistical analyses were performed with GraphPad Prism version 6.0e using one-way analysis of variance (ANOVA) or, for the neuraminidase inhibition titration, two-way ANOVA, both with Sidak's multiple-comparison tests. Statistical significance was demonstrated by a *P* value of <0.05.
